# RE: A step-by-step guide for remote working in the NHS: evaluation of a virtual consultant psychiatrist hiring scheme

**DOI:** 10.1192/bjb.2025.10146

**Published:** 2025-10

**Authors:** Dania Mann-Wineberg, Rahul Tomar

**Affiliations:** FY1 Doctor, Hertfordshire Partnership Foundation Trust, UK; Consultant Psychiatrist, Hertfordshire Partnership Foundation Trust, UK

## Are virtual consultant hiring schemes in the best interests of patients and clinicians?

I was intrigued by the concept of an entirely remote psychiatry consultant position outlined in ‘A step-by-step guide for remote working in the NHS: evaluation of a virtual consultant psychiatrist hiring scheme’.^1^ I work as a resident doctor in old age psychiatry, and although virtual consultations seem to have become an inevitable part of the future in the aftermath of the COVID-19 pandemic,^2^ it seems that the conclusions in this article present some challenges.

An initiative that was trialled in one mental health trust^3^ provides reflective points regarding recruitment for remote psychiatry consultants as a post-pandemic response. Feedback after remote memory clinic consultations was largely positive, although patients expressed a preference for face-to-face consultations. The role of the consultant here was entirely remote, relying on a specialist nurse being present with the patient in person. Perhaps filling positions with virtual consultants could increase the burden on those who work face-to-face, as well as putting more pressure on allied healthcare professionals, risking burnout.

Another issue is that of a consultant psychiatrist who has never seen the patient relying on reports from a nurse who does not have the same category of medical training. This could be dangerous, for example, where a patient presents to memory clinic with signs of dementia, but a physical examination from someone who knows which signs to look for would reveal bradykinesia, rigidity and a resting tremor, all of which could be missed, leading to a diagnosis of Parkinson’s disease or Lewy body dementia^4^ being overlooked.

Finally, with fewer in-person interactions being cited as contributing to poorer mental health,^5^ perhaps there is irony to decreasing the face-to-face nature of psychiatric consultations. It could certainly be argued from the above that initiatives such as these will affect the most vulnerable patients disproportionately.

There is a clear necessity to fill roles and utilise developments in technology, and Havard et al^1^ approached the issue with these factors in mind. Perhaps new technological advances in virtual reality and artificial intelligence could negate some of the above concerns and help to deliver hybrid services that are beneficial for patients and clinicians alike.
